# Use of Pharmacokinetic Modeling to Design Studies for Pathway-Specific Exposure Model Evaluation

**DOI:** 10.1289/ehp.6367

**Published:** 2004-08-16

**Authors:** Ye Hu, Gerry G. Akland, Edo D. Pellizzari, Maurice R. Berry, Lisa Jo Melnyk

**Affiliations:** ^1^Analytical and Chemical Sciences, Research Triangle Institute, Research Triangle Park, North Carolina, USA; ^2^National Exposure Research Laboratory, U.S. Environmental Protection Agency, Cincinnati, Ohio, USA

**Keywords:** dietary intake, exposure, pesticide, pharmacokinetic (PK) modeling, study design

## Abstract

Validating an exposure pathway model is difficult because the biomarker, which is often used to evaluate the model prediction, is an integrated measure for exposures from all the exposure routes and pathways. The purpose of this article is to demonstrate a method to use pharmacokinetic (PK) modeling and computer simulation to guide the design of field studies to validate pathway models. The children’s dietary intake model is discussed in detail as an example. Three important aspects are identified for a successful design to evaluate the children’s dietary intake model: *a*) longitudinally designed study with significant changes in the exposure for the route/pathway of interest, *b*) short biologic half-life of the selected chemical, and *c*) surface loading of the selected chemical at sufficient levels. Using PK modeling to guide a study design allowed a path-specific exposure model to be evaluated using urinary metabolite biomarkers.

Modeling is often the only cost-effective tool for making exposure and risk assessments; however, an evaluation of such modeling is difficult, especially if it is for a pathway-specific model such as a dietary exposure model. A biomarker, such as urinary metabolite, which is often used to evaluate a model prediction, is an integrated measure of exposures from all routes, including inhalation, ingestion, and dermal. Biomarkers also have inherent problems such as large intra- and interindividual variabilities and unclear metabolic pathways. These uncertainties complicate the interpretation of biomarker measurements relative to the routes responsible for the exposures. Furthermore, the detection limits for urinary metabolite biomarkers are often not low enough to obtain a measurement, producing a substantial number of nonmeasurable observations, which make model validation impossible.

Despite these problems, the demand for model evaluation is increasing (Oreskes 1998). Biomarkers have been used to evaluate various exposure models, such as a lead exposure model (Zaragoza and Hogan 1998), a dietary cadmium model (Choudhury et al. 2001), and a dietary methyl mercury intake model (Ponce et al. 1998). In all these studies, however, pharmacokinetic (PK) modeling was used to provide interpretations for exposure and biomarker measurement. The potential of PK modeling in guiding a study design for model evaluation was not explored.

One of the problems in children’s exposure studies is assessing dietary exposure. Because children touch foods with their hands, excess dietary intake could result from hand-to-food, surface-to-food, and hand-to-surface-to-food contacts in contaminated homes (Melnyk et al. 2000). No direct method to measure this excess exposure is available, so a dietary intake model was developed (Akland et al. 2000).

Because the children’s dietary intake model is pathway specific, evaluating it has considerable challenges. PK modeling makes the evaluation possible. Unlike other model evaluation efforts, here we used PK modeling to guide the design of a field study to evaluate a pathway-specific model using urinary metabolites measured in overnight voids. The children’s dietary intake model for pesticide exposure is used as an example. The principle of using PK modeling for study design, however, should be applicable in other similar cases.

## Materials and Methods

### Conceptual model.

**Figure 1:**
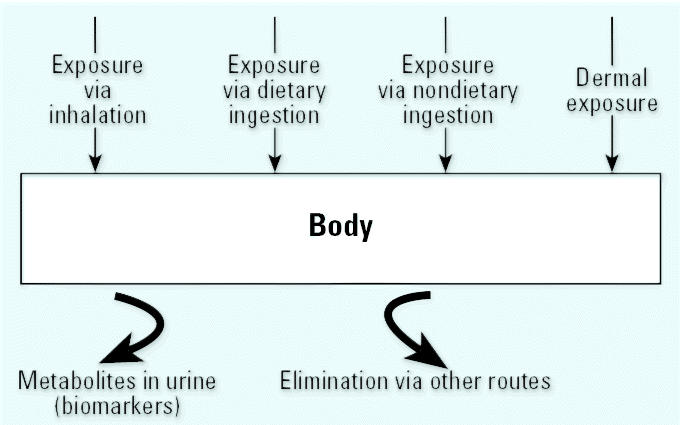
Single-compartment model for exposures from different pathways.

A simplified, single-compartment model that can be used in the design of a field study is shown in Figure 1. In a single-compartment model, the body receives exposures from three major routes: inhalation, ingestion, and dermal. The ingestion route receives exposures from two pathways: dietary ingestion and nondietary ingestion (caused by hand-to-mouth or object-to-mouth activities). The body eliminates the exposure through urine and other biologic routes, such as exhaled air, feces, and other body fluids.

**Figure 2:**
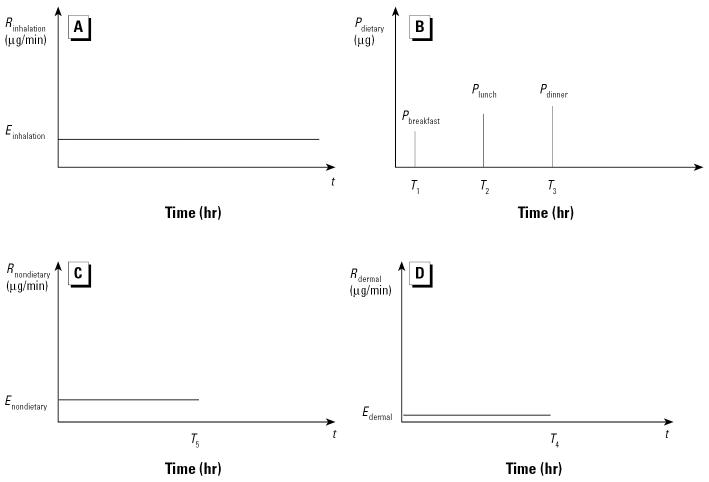
Exposure functions for a hypothetical child. (*A*) Hypothetical inhalation exposure. (*B*) Hypothetical dietary intake. (*C*) Hypothetical nondietary exposure. (*D*) Hypothetical dermal exposure.

To demonstrate how a specific pathway model can be evaluated using an overnight urine void, a hypothetical scenario (shown in Figure 2) is presented here. In this hypothetical case, a child receives discrete and varying amounts of dietary exposure, *P*_dietary_ (micrograms), from the meals (see Table 1 for a list of all terms used in this article). A child also receives a simplified constant rate for inhalation exposure, *R*_inhalation_ (micrograms per minute), assuming the child spends most of the time indoors (Lambert et al. 1993). In addition, the child receives a fairly constant nondietary ingestion exposure, *R*_nondietary_ (micrograms per minute), from hand-to-mouth or object-to-mouth activities that occur when the child is awake during the day. Finally, the child receives a constant rate of dermal exposure, *R*_dermal_ (micrograms per minute), during the day until he or she is bathed. The exposure amount and rates can be expressed as follows:


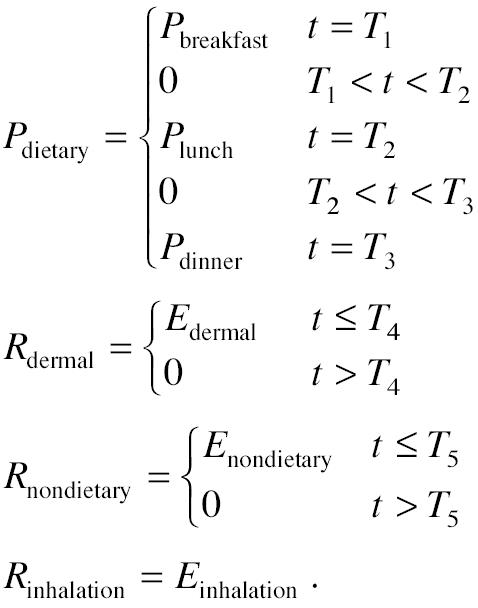


Here *P*_breakfast_, *P*_lunch_, and *P*_dinner_ are the amount of dietary intake from breakfast, lunch, and dinner, respectively, and *T*_1_, *T*_2_, and *T*_3_ are the timing of the meals. *E*_dermal_ is the rate of dermal exposure before bathing, and *T*_4_ is the time when the child is bathed. *E*_nondietary_ is the rate of nondietary exposure before bed, and *T*_5_ is the time when the child goes to the bed. *E*_inhalation_ is the constant rate of inhalation exposure.

Assuming immediate and 100% absorption through all routes for a single-compartment linear model, the change in the amount of pollutant over time in the compartment can be expressed as follows:


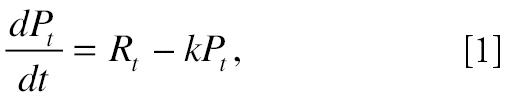


where *P_t_* is the amount of pollutant in the compartment, and *k* is the first-order biologic elimination constant, calculated by 0.693/*T*_1/2_ (*T*_1/2_ is the biologic half-life) (Schoenwald 2001). *R_T_* is the sum of *R*_inhalation_, *R*_nondietary_, and *R*_dermal_. Dietary exposures from the three meals can be viewed as additional multiple bolus intake at times *T*_1_, *T*_2_, and *T*_3_.

Using the principle of superposition (Schoenwald 2001), the solution to Equation 1 can be expressed as follows:


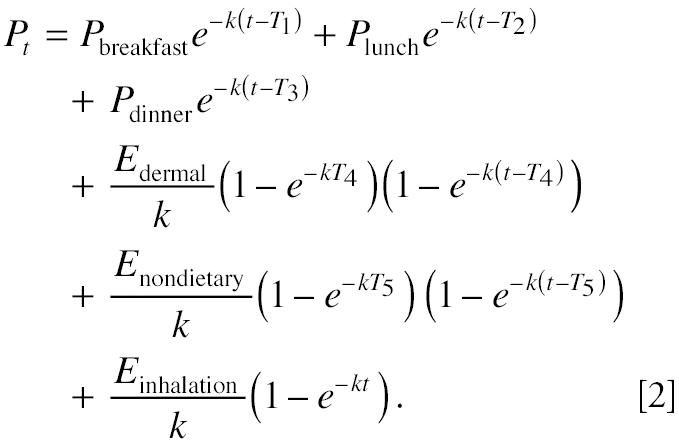


The amount of pollutant metabolite eliminated into overnight void from 2000 hr to 0800 hr is:


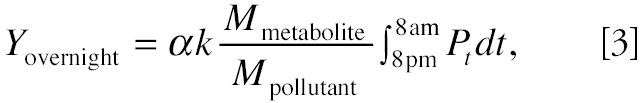


where α is the fraction of pollutant that is eliminated via urine, *k* is the first-order biologic elimination constant, *P_t_* is the amount of pollutant in the compartment, *M*_pollutant_ is the molecular weight of the pollutant, and *M*_metabolite_ is the molecular weight of the urinary metabolite. Applying Equation 2 to Equation 3, the amount of metabolite in overnight urine, *Y*_overnight_, becomes


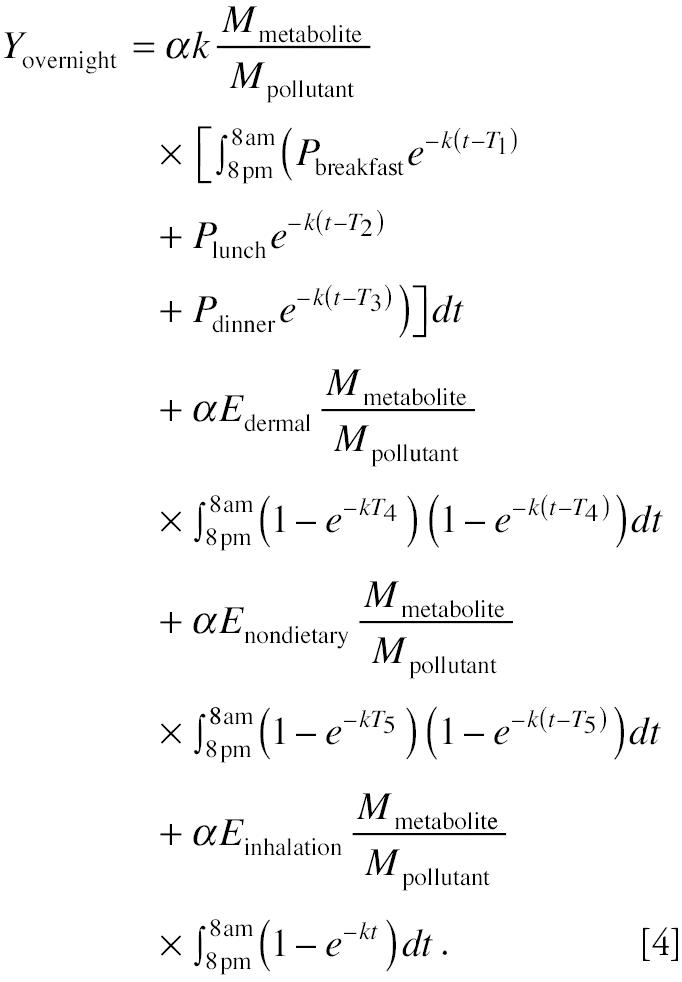


Equation 4 demonstrates that the amount of metabolite in overnight urine is an additive result of exposure from dietary ingestion, nondietary ingestion, inhalation, and dermal exposure. Therefore, if we design a study in which exposure from a specific route is varied while exposures from other routes remain the same, we will be able to investigate the exposure through this particular route. For example, if we alternate only daily dietary exposure status—that is, let the subject have dietary exposure on “dietary exposure day” (when dietary exposures are *P*_breakfast_, *P*_lunch_, and *P*_dinner_) followed by “no dietary exposure day” (when dietary exposures *P*_breakfast_ = *P*_lunch_ = *P*_dinner_ = 0)—and let the exposures from other routes/pathways remain the same, then the difference of the urinary metabolites between these 2 days is a function only of dietary exposure because exposures from other routes/pathways can be canceled out. Equation 5 shows the difference in the amount of urinary metabolites measured in overnight voids after the dietary-exposure day and the no-dietary-exposure day:


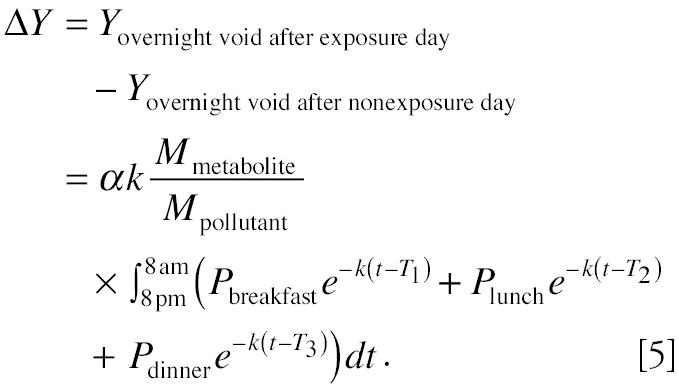


Equation 5 indicates that if Δ*Y*—the metabolite difference between overnight voids after the dietary-exposure day and the no-dietary-exposure day—is large enough to be measured, it can be used to evaluate dietary exposure differences on these days. It also indicates that to make the evaluation possible, the dietary exposures on the dietary-exposed day also need to be large; the biologic half-life of the chemical, *T*_1/2_, needs to be short because *k* is proportional to 1/*T*_1/2_; and a substantial fraction of the metabolites should be eliminated through the urinary pathway.

In reality, however, dietary exposure is hardly zero on the dietary exposure days, because pesticide residues in foods are inevitable. Nonetheless, with a careful design, the pesticide residue can be canceled out and the strategy can still be used, as demonstrated in the following evaluation of the children’s dietary intake model.

### Children’s dietary intake model.

The major problem of assessing children’s dietary exposure is that young children often touch foods with their hands before consumption, thereby increasing contamination of the food and their intake of contaminants through the diet (Melnyk et al. 2000). Because direct methods for sampling the foods as they enter the mouths of young children are not available, a deterministic dietary intake model was developed (Akland et al. 2000). In this model, three terms are considered: *a*) the original contaminant residue on the food before handling (term 1), *b*) surface-to-food contamination as the food comes in contact with contaminated surfaces (term 2), and *c*) surface-to-hand-to-food contamination as the child touches the contaminated surfaces and then handles and eats foods (term 3). Term 1 has also been referred to as “direct dietary ingestion,” and terms 2 and 3 as “indirect dietary ingestion.” In this model, it is assumed that the activity parameters (*A_S_*_/_*_F_*, *A_H_*_/_*_F_*, and *A_S_*_/_*_H_*) are determined by food types and individual child, and transfer efficiencies (*T_S_*_/_*_F_*, *T_H_*_/_*_F_*, and *T_S_*_/_*_H_*) are determined by food types, surface types, and the chemical properties of the contaminants.

Details of the children’s dietary intake model have been discussed previously (Akland et al. 2000). The following is the model for a specific food item consumed after multiple touches by hands and/or surfaces.


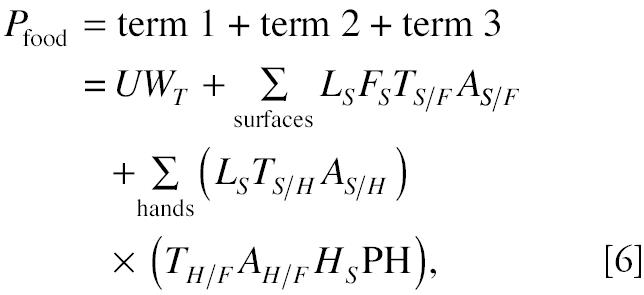


where, assuming the pollutant of interest is a pesticide, *P*_food_ is the dietary intake of a pesticide for one food (micrograms), *U* is the pesticide residue concentration (micrograms pesticide per gram food), *W_T_* is the total amount of the individual food consumed (grams), *L_S_* is the loading of the contaminant on the surface (micrograms pesticide per square centimeter), *F_S_* is the food surface area that comes in contact with the contaminated surface (square centimeters), *T_S/F_* is the surface-to-food mass transfer efficiency (dimensionless), *A_S_*_/_*_F_* is the surface-to-food contact frequencies, *T_S/H_* is the surface-to-hand mass transfer efficiency (dimensionless), A*_S/H_* is the surface-to-hand contact frequencies, T*_H/F_* is the hand-to-food mass transfer efficiency (dimensionless), A*_H/F_* is the hand-to-food contact frequency, *H_S_* is the total hand surface area (square centimeters), and *PH* is the proportion of hand surface area in contact with contaminated food. Total dietary exposure for a meal is therefore


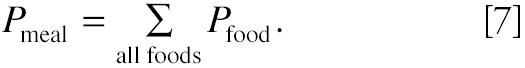


Laboratory experiments have demonstrated measurable surface-to-food, surface-to-hand, and hand-to-food pesticide transfers (Akland et al. 2000; Edwards and Lioy 1999). Using the children’s dietary intake model Equation 6, Akland et al. (2000) estimated that the extra pesticide intake resulting from young children’s eating behaviors, terms 2 and 3, could account for up to 80% of total dietary intake if the surface loading of pesticide residue is 5 ng/cm^2^ or higher (Akland et al. 2000).

If proved, this result would have profound implications in pesticide regulation and exposure mitigation. However, as shown in Equation 6, the model prediction was based upon the estimation of food surfaces, the surface pesticide loading, the transfer efficiencies, and observation of children’s eating behaviors. A natural question for the model prediction is whether this model estimation is reasonable.

### Using PK modeling to design a field study: children’s dietary intake model as an example.

#### General concept for design.

The children’s dietary intake model is a pathway model. Exposures from other routes/pathways (e.g., nondietary ingestion, inhalation, and dermal exposure) also contribute to the total urinary pesticide metabolite measurements. Therefore, using urinary biomarker measurements to evaluate the dietary intake model is difficult. To circumvent the problem, the strategy demonstrated in Equation 5 can be followed, as outlined below.

According to the children’s dietary intake model Equation 6, the dietary exposure consists of three terms: residue in food before handling (term 1), surface-to-food transfer (term 2), and surface-to-hand-to-food transfer (term 3). On a day when the child is allowed to eat in an unrestricted normal setting, the child receives environmental exposures through inhalation, dietary ingestion, nondietary ingestion, and dermal exposure, and the dietary exposure includes term 1 + term 2 + term 3. Suppose we restrict a child with clean hands to a clean area and require the same foods to be eaten as on the normal day; then term 2 + term 3 are artificially forced to be approximately zero and only term 1 remains. For the convenience of discussion, henceforth the day when the child is restricted to a clean area with clean hands is referred to as “nonexposed day,” and the day when the child is allowed to eat at regular places with uncleaned hands is referred to as “exposed day.” Note that on the nonexposed day, the child still receives inhalation, nondietary ingestion, and dermal exposures. On both the exposed day and the nonexposed day, the child receives the same term 1 because the same foods are eaten on both days. The exposures the child does not receive on the nonexposed day are the surface-to-food transfer (term 2) and surface-to-hand-to-food transfer (term 3).

Theoretically, if inhalation, nondietary ingestion, and dermal exposures can be kept approximately the same on the exposed day and the nonexposed day, then according to Equation 5, the difference in the amount of urinary metabolites in overnight voids after the exposed day and the nonexposed day is a function of terms 2 and 3:


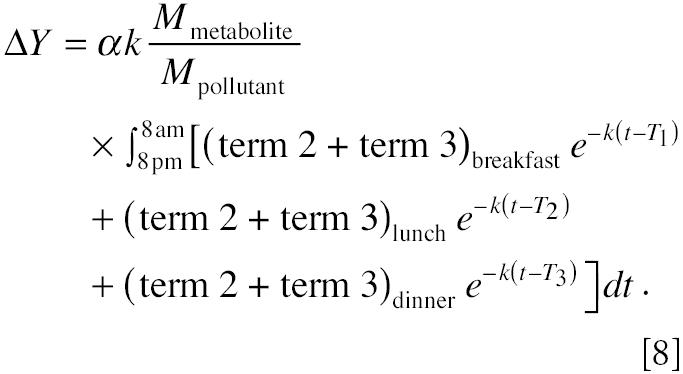


Compared with Equation 5, term 1 has been canceled out because the child’s diet is restricted so that the same foods were eaten on the exposed day and the nonexposed day. An effective method to maintain the same exposure on the exposed day and the nonexposed day for other exposure routes/pathways while alternating the exposure for the pathway of interest is to conduct the study longitudinally so that data from several exposed-day/nonexposed-day pairs can be collected from the same subjects. This way the participant can serve as his or her own control so that α and *k* can be assumed to be the same variable and behavior pattern variations can be kept at a minimum.

#### Computer simulation.

Equations 5 and 8 demonstrate how, in theory, a route/pathway exposure model can be evaluated with a study design using metabolites in overnight urinary voids where the exposure status of the route/pathway of interest is varied while the exposures from the other routes/pathways are kept the same. For field studies, the following questions are the keys for study design: How long should the half-life of a selected pesticide be? What is the minimum level of surface pesticide loading to produce a measurable metabolite concentration in the overnight void? What is the minimum level of surface pesticide loading to make indirect dietary ingestion a measurable quantity in overnight urine? Will exposures from other pathways “mask” the exposure caused by surface-to-food and surface-to-hand-to-food transfer? How large a sample size is needed?

An important assumption for the analytical solutions, Equations 5 and 8, is that exposures from inhalation, nondietary ingestion, and dermal remain constant. In reality, however, this may not be true. To investigate whether a varying inhalation–nondietary–dermal profile will mask the urinary metabolite difference caused by dietary exposure, which is the key to the study design, we need to let the exposure rates vary across time. To demonstrate, however, we only set nondietary ingestion exposure to vary across time because of its significance (Zartarian et al. 2000). Inhalation and dermal exposures remained constant.

The varying exposure rates make it impossible to use analytical solutions to Equations 5 and 8. Therefore, we conducted a computer simulation to answer the above questions needed for a field study. To conduct the computer simulation, we set all the input parameters at values for a likely scenario based upon published literature. The parameters of interest were then varied (one at a time) to observe their impact on the output variable (i.e., urinary metabolite concentration). Computer simulation was based upon numerical solution to Equation 3 using Euler’s method (Grossman 1986):


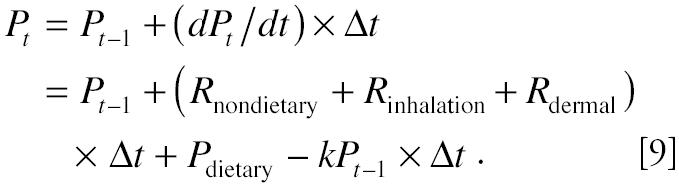


Details of the estimation/simulation of the exposure rates are given below.

For the inhalation exposure rate, exposure via inhalation per hour was estimated as follows:


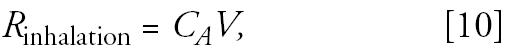


where *C_A_* is the air concentration (micrograms per liter) and *V* is the ventilation rate for children (liters per hour).

The nondietary ingestion exposure rate mentioned here is the exposure incurred when children put contaminated hands or toys into their mouth. To simulate the varying profile, the time that a child is awake (assuming from 0800 hr to 2000 hr) was divided into equal time intervals. The nondietary exposures received in these time intervals were assumed to be normally distributed. The mean of the *R*_nondietary_ was calculated by the following formula:


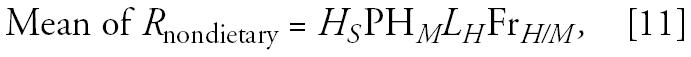


where *H_S_* is the total hand/toy surface area (square centimeters), PH*_M_* is the proportion of total hand/toy surface area coming in contact with mouth, *L_H_* is the surface loading of the contaminant on the hand/toy (micrograms pesticide per square centimeter), and Fr*_H/M_* is the frequency of mouthing activity during the time interval. Using published data, we estimated a mean of 0.0267 μg/min for *R*_nondietary_. A standard deviation of 0.0179 μg/min was assumed so that > 50% of the simulated values were within one standard deviation (Table 2). Because nondietary ingestion exposure was unlikely when the child is asleep, we assumed zero nondietary exposures between 2000 hr and 0800 hr. The simulation of normally distributed *R*_nondietary_ for a 1-min time interval can be summarized in the following formula:


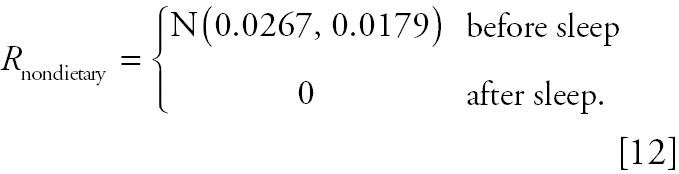


We ignored dermal exposure in the computer simulation for two reasons. First, exposure to diazinon (which was the pesticide of interest) through skin absorption has been reported in the literature to be minimal, although this may not be the case for other chemicals. Using radiolabeled diazinon in an acetone solution or lanolin grease on the forearm or abdomen, Wester et al. (1993) reported a total of only 2.2% skin absorption over 24 hr. Second, the purpose of the study was to guide study design rather than to establish a definitive relationship between exposure and metabolites.

Applying Equations 7, 10, and 12 to Equation 9, the model used to conduct the computer simulation was obtained. Table 2 lists the parameters used to estimate inhalation and nondietary intake.

Parameters for the children’s dietary intake model were obtained from a previous study (Akland et al. 2000). Table 3 demonstrates how to use the children’s dietary model to estimate exposure for three example foods: Cheerios, apple, and tortilla. In these examples, the pesticide residue was assumed to be 6 ng/g for all foods (National Research Council 1993). Parameters *T_S/H_*, *A_S/H_*, *T_H/F_*, *A_H/F_*, and PH were also estimated from the previous study (Akland et al. 2000). Because Cheerios are normally eaten with utensils, only term 1 is calculated for total dietary ingestion. Apple and tortilla, however, were estimated for terms 2 and 3, because these foods are normally handled by children. Other foods used to estimate a hypothetical child’s exposed day’s total dietary intake included rice (two tablespoons), chicken nuggets (four pieces), and ham (one slice). On the next unexposed day, only term 1 from the foods remained, and terms 2 and 3 were assumed to be zero. The examples shown in Table 3 demonstrate that by varying surface loading, different pesticide transfers are obtained. Therefore, the minimum level of surface pesticide loading to make indirect dietary ingestion a measurable quantity in overnight urine can be estimated.

Computer simulation was conducted using Microsoft Excel 2002 (Microsoft, Seattle, WA). Equations for calculating *R*_inhalation_, *R*_nondietary_, and *P*_dietary_ were keyed in, and variables of interest, such as biologic half-life, dust loading, air concentration, and nondietary intake, were set in such a way that they could be easily varied to conduct the simulation. The simulation results were also plotted using Microsoft Excel.

### Sample size calculation.

Once the results from the simulation were obtained, sample size was calculated based upon a one-sided *t*-test of hypothesis: *Y*_overnight void after exposed day_ = *Y*_overnight void after nonexposed day_ versus *Y*_overnight void after exposed day_ > *Y*_overnight void after nonexposed day_ (Kleinbaum et al. 1988).

## Results

### Urinary measurements and biologic half-life.

**Figure 3:**
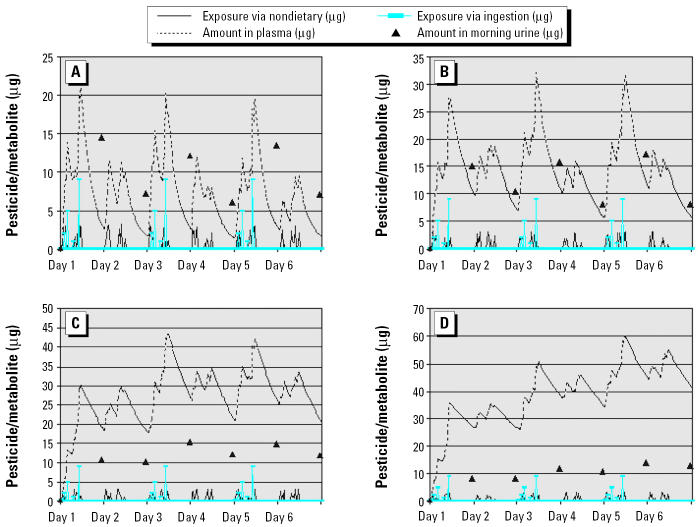
Effect of biologic half-life on urinary measurements in the nonexposed-day/exposed-day design. (*A*) Half-life = 4 hr. (*B*) Half-life = 8 hr. (*C*) Half-life = 16 hr. (*D*) Half-life = 27 hr.

Figure 3 shows the urinary metabolite measurements in overnight voids as point estimates (when the urine samples are collected at 0800 hr) after three exposed-day/nonexposed-day pairs with various lengths of biologic half-life of the selected chemical. The results indicated that the success of the validation depends heavily on the biologic half-life of the chosen chemical. If the chemical has a relatively short half-life, as does malathion (3–4 hr; Lyon et al. 1987) or diazinon (~ 6 hr; Iverson et al. 1975), it is possible to detect a change in the urine metabolite concentration. The amount in the plasma also returns to nonexposed levels, which makes the evaluation of the model possible. However, if the biologic half-life is longer than 16 hr, a large sample size is required because the difference between urinary metabolites after exposed days and nonexposed days becomes small and the amount in the plasma is carried over from day to day with no recovery. When the biologic half-life is as long as or longer than 27 hr (e.g., chlorpyrifos), the chance of successful validation using the exposed-day/nonexposed-day design is even smaller because there is minimal difference in the urinary metabolite concentrations. Nonetheless, an alternative design, such as 1 exposed day followed by 2 nonexposed days to let the body further eliminate the metabolites, might be possible. This alternative design, however, substantially increases field difficulties because on the 2 non-exposed days the field team would need to ensure that no term 2 or term 3 intakes occur.

### Pesticide loading.

**Figure 4:**
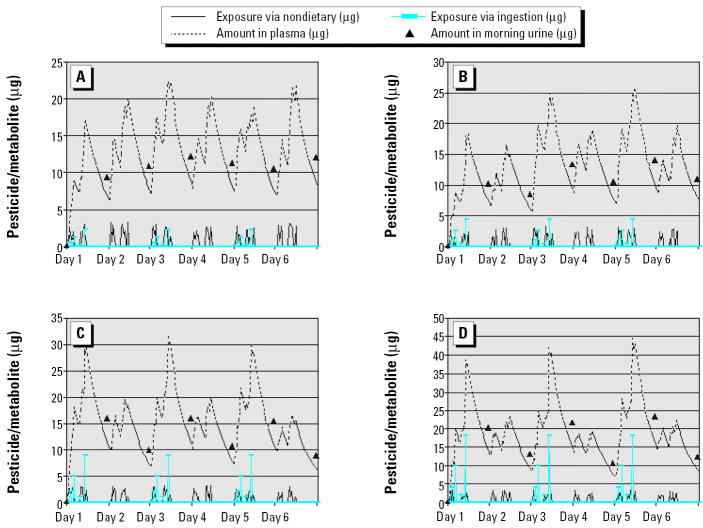
Effect of surface loading on urinary metabolite measurements in the nonexposed-day/exposed-day design. (*A*) Surface loading = 1 ng/cm^2^. (*B*) Surface loading = 2 ng/cm^2^. (*C*) Surface loading = 4 ng/cm^2^. (*D*) Surface loading = 8 ng/cm^2^.

Surface pesticide loading is the source for surface-to-food and surface-to-hand-to-food transfer. Results of variations in the surface loading and urinary metabolites for a compound with a biologic half-life of 8 hr are shown in Figure 4. The results indicate that even if the chemical’s half-life is short, a preferable loading of 4 ng/cm^2^ or above is still needed to generate observable differences in urinary metabolites in the overnight voids after the exposed day and the nonexposed day. This level of loading can be found after indoor pesticide application (Byrne et al. 1998). However, when the loading decreases to ≤ 1 ng/cm^2^, it is very difficult to see the differences in the urinary metabolite amount in overnight voids after exposed and nonexposed days. In the Minnesota Children’s Pesticide Exposure Study, the mean surface chlorpyrifos loading measured by a surface press ranged from 0.03 to 32.6 ng/cm^2^, with a mean of 0.48 ng/cm^2^ (Lioy et al. 2000). These results answer the question about exposure scenario: Households with surface pesticide loading > 4 ng/cm^2^ are preferred for efficient design, and houses that have frequent indoor pesticide applications are most likely to meet the criterion.

### Impact of exposure from other routes/pathways.

**Figure 5:**
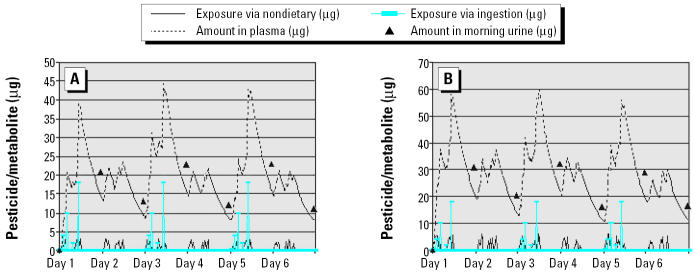
Effect of nondietary exposure on urinary measurements in the nonexposed-day/exposed-day design. (*A*) Nondietary ∼ normal distribution, mean ± SD = 0.0266 ± 0.0179. (*B*) Nondietary ∼ normal distribution, mean ± SD = 0.0532 ± 0.0258.

Figure 5 attempts to answer whether exposure from nondietary ingestion will mask the dietary exposure and interfere with the validation process. As shown in Figure 5, when nondietary ingestion exposure is normally distributed with a mean ± SD of 1.6 ± 1.1 μg/hr, the mask effect is small enough to allow the biomarker differences caused by dietary exposure difference to be observed. However, when nondietary ingestion exposure reaches a mean of 3.2 μg/hr, the mask effect becomes obvious because the difference in urine metabolite concentrations becomes small and inconsistent. The 1.6 μg/hr nondietary ingestion exposure was calculated by assuming a mouthing frequency of 10/hr (Reed et al. 1999; Zartarian et al. 1997), which was high compared with the current U.S. Environmental Protection Agency default (Reed et al. 1999), and for each event the child mouths a 40-cm^2^ surface (hand or toy) with a relatively high pesticide loading of 4 ng/cm^2^. Because these assumptions reflect high-end exposure, we can safely assume that the average level of nondietary activity will not significantly interfere with the model validation process. Nonetheless, to conduct a successful study, the subjects selected into the study would preferably be children who do not have frequent mouthing activities, such as thumb sucking.

**Figure 6:**
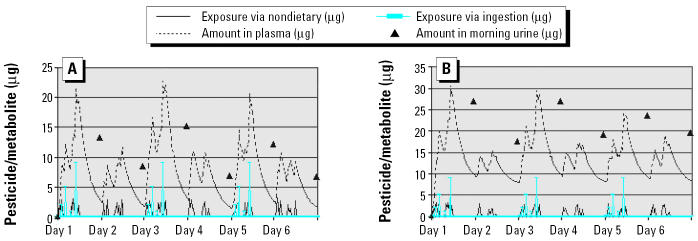
Effect of inhalation exposure on urinary metabolite measurements in the nonexposed-day/exposed-day design. (*A*) Air concentration = 0.5 μg/m^3^. (*B*) Air concentration = 5 μg/m^3^.

Similarly, we estimated the effect of inhalation exposure (Figure 6). The results indicate that inhalation exposure does not cause a large effect on the biomarker differences, even when the hypothetical air concentration was increased to 5 μg/m^3^, a level only seen immediately after indoor pesticide application (Akland et al. 2000).

### Sample size.

Based upon a pesticide with a biologic half-life of 8 hr and assuming a variance of 2 due to measurement errors, a minimum sample size of five pairs of the exposed day and the nonexposed day would be required in homes with pesticide loading ≥ 4 ng/cm^2^ to achieve a power of 80% for detecting 3-μg urinary metabolite differences.

## Discussion

Evaluating a pathway model is difficult because the biomarker measurements also have contributions from other exposure routes/pathways. Here we demonstrate that a thoughtful design guided by PK modeling can make the evaluation possible. The computer simulation for the children’s dietary intake model indicated three important aspects for a successful design: longitudinal design of the study, short half-life of the selected chemical, and high pesticide surface loading. Under normal circumstances, inhalation and nondietary ingestion exposure would not mask the dietary exposure as long as they can be kept nearly constant for the nonexposed day and the exposed day.

Using the results from the computer simulation, we selected diazinon and conducted a study with three children in homes with surface loading of > 4 ng/cm^2^. Each child was followed for at least 6 days, yielding three or more nonexposed-day/exposed-day pairs. The study results (unpublished data) indicated that this design was successful. Using PK modeling as a guidance, field efforts to collect data to evaluate the model can be well planned, and the cost can be substantially reduced.

In this study, we used a single-compartment PK model. The single-compartment model may not be as accurate as a multicompartment PK model in prediction, but it has a practical advantage—only two parameters are essential to build a model: the biologic half-life of the chemical and the proportion of the chemical eliminated in overnight void. In many cases, these parameters are the only information one can obtain from the literature. Because of this practical advantage, the single-compartment model was recently used again by other researchers to assess pesticide exposure based on urinary biomarkers (Rigas et al. 2001). Because the purpose of this modeling approach is to provide guidance for the design of field studies, it is perhaps not necessary to expend large efforts to develop a complicated model at the front end of the study design. Our field study also indicated that the single-compartment model was adequate for designing the model evaluation study we had conducted.

This article demonstrated the case of designing a study to appropriately capture data in order to evaluate a dietary exposure model. However, we envision a similar strategy being used in other situations, such as nondietary ingestion exposure models, dermal exposure models, or inhalation exposure models.

## Figures and Tables

**Table 1:** Notations.

Abbreviation	Definition
α	Fraction of pollutant that is eliminated through urine
*A*_*H/F*_	Hand-to-food contact frequencies
*A*_*S/F*_	Surface-to-food contact frequencies
*A*_*S/H*_	Surface-to-hand contact frequencies
*A*_*H/M*_	Hand (toy)-to-mouth contact frequencies
*C*_*A*_	Air concentration (μg/L)
*Fr*_*H/M*_	Frequency of mouthing activity during a time interval of interest
*F*_*S*_	Food surface area that comes in contact with the contaminated surface (cm^2^)
*H*_*S*_	Total hand surface area (cm^2^)
*k*	First-order elimination rate constant
*L*_*H*_	Loading of contaminant on hand/toy (μg contaminant/cm^2^)
*L*_*S*_	Loading of contaminant on surface (μg contaminant/cm^2^)
*M*_metabolite_	Molecular weight of urinary metabolite
*M*_pollutant_	Molecular weight of pollutant compound
*P*_breakfast_	Amount of pollutant in breakfast (μg)
*P*_lunch_	Amount of pollutant in lunch (μg)
*P*_dinner_	Amount of pollutant in dinner (μg)
*P*_food_	Amount of pollutant in one food (μg)
*P*_meal_	Amount of pollutant in one meal (μg)
*P*_dietary_	Amount of dietary exposure received from all meals (μg)
*PH*	Proportion of hand surface area in contact with contaminated food
*PH*_*M*_	Proportion of total hand/toy surface area coming in contact with mouth
*P*_*t*_	Amount of pollutant in the compartment (μg)
*R*_dermal_	Dermal exposure rate (μg/hr)
*R*_inhalation_	Inhalation exposure rate (μg/hr)
*R*_nondietary_	Nondietary ingestion exposure rate (μg/hr)
*R*_*T*_	Sum of *R*_internal_, *R*_inhalation_, and *R*_nondietary_
*T*_1_	Timing for breakfast
*T*_2_	Timing for lunch
*T*_3_	Timing for dinner
*T*_4_	Timing for bath
*T*_5_	Timing when child goes to bed
*T*_*H/F*_	Hand-to-food transfer efficiencies
*T*_*S/F*_	Surface-to-food transfer efficiencies
*T*_*S/H*_	Surface-to-hand transfer efficiencies
*U*	Pollutant residue in food (μg/g)
*V*	Ventilation rate for children (L/hr)
*W*_*T*_	Total amount of food consumed (g)
*Y*_overnight_	Amount of urinary metabolite in overnight void
*Y*_overnight void after exposure day_	Amount of urinary metabolite in overnight void after exposed day
*Y*_overnight void after nonexposure day_	Amount of urinary metabolite in overnight void after nonexposed day

**Table 2:** Parameters for inhalation and nondietary ingestion exposures.

	Type of distribution used in simulation	Variable (reference)
Inhalation exposure	Constant	*V* = 4.2 L/min
		*C*_*A*_ = 0.5 μg/m^3^ (Byrne et al. 1998)
Nondietary exposure	Normal distribution with mean ± SD = 0.0267 ± 0.1795 μg/min for 0800–2000 hr; 0 for 2000–0800 hr	*H*_*S*_ = 200 cm^2^
		PH_*M*_ = 0.2
		*L*_*H*_ = 4 ng/cm^2^ (Byrne et al. 1998; Lu and Fenske 1999)
		Fr_*H/M*_ = 10/hr (Reed et al. 1999; Zartarian et al. 1997)

**Table 3:** Parameters used to calculate dietary intake from Cheerios, apple, and tortilla (Akland et al. 2000).

Parameters	Parameter values	Dietary intake
Cheerios (half bowl)		
Term 1*^a^*		
*R*	0.006 μg/g	
*F*_*T*_	30 g	Term 1 = 0.18 μg
		*P*_breakfast_ = term 1 = 0.18 μg
Apple (1/3 apple)		
Term 1		
*R*	0.006 μg/g	
*F*_*T*_	80 g	Term 1 = 0.48
Term 2*^b^*		
*F*_*S*_	100 cm^2^	
*L*_*S*_	0.004 μg/cm^2^	
*T*_*S/F*_	0.5	
*A*_*S/F*_	1	Term 2 = 0.2
Term 3*^c^*		
*L*_*S*_	0.004 μg/cm^2^	
*T*_*S/H*_	0.4	
*A*_*S/H*_	10	
*T*_*H/F*_	0.03	
*A*_*H/F*_	10	
*H*_*S*_	200 cm^2^	
*PH*	0.9	Term 3 = 0.86
		*P*_lunch_ = term 1 + term 2 + term 3 = 1.54
Tortilla (half of a tortilla)		
Term 1		
*R*	0.006 μg/g	
*F*_*T*_	65 g	Term 1 = 0.39
Term 2		
*F*_*S*_	200 cm^2^	
*L*_*S*_	0.004 μg/cm^2^	
*T*_*S/F*_	0.5 (chair-food)	
*A*_*S/F*_	1	Term 2 = 0.4
Term 3		
*L*_*S*_	0.004 μg/cm^2^	
*T*_*S/H*_	0.5	
*A*_*S/H*_	20	
*T*_*H/F*_	0.03	
*A*_*H/F*_	20	
*H*_*S*_	200 cm^2^	
*PH*	0.9	Term 3 = 4.32
		*P*_dinner_ = term 1 + term 2 + term 3 = 5.11

Using model Equation 10 to estimate dietary intake for apple.

a Term 1 = 0.006 (μg/g) × 30 (g) = 0.18 μg.

b Term 2 = 100 (cm^2^) × 0.004 (μg/cm^2^) × 0.5 × 1 = 0.2 μg.

c Term 3 = 0.004 (μg/cm^2^) × 0.4 × 10 × 0.03 × 10 × 200 (cm^2^) × 0.9 = 0.86 μg.
